# Reply to H Daungsupawong and V Wiwanitkit

**DOI:** 10.1016/j.psj.2025.105423

**Published:** 2025-06-12

**Authors:** Ewa M. Drzewiecka, Ewa Liszewska, Krzysztof Kozłowski, Andrzej Ciereszko, Mariola Słowińska

**Affiliations:** a Team of Gamete Biology, Institute of Animal Reproduction and Food Research, Polish Academy of Sciences in Olsztyn, Trylińskiego 18 10-683, Olsztyn, Poland; b Department of Poultry Science and Apiculture, Faculty of Animal Bioengineering, University of Warmia and Mazury in Olsztyn, Oczapowskiego 5 10-719, Olsztyn, Poland

Dear Editor,

Recently, we have read the letter to the editor by Daungsupawong H. and Wiwanitkit V. on our recent paper “Reference genes selection for gene expression analyses in reproductive turkey (*Meleagris gallopavo*) with yellow semen syndrome" (Drzewiecka et al., 2025) published in Poultry Science (Volume 104, Issue 7, July 2025, 105182).

We appreciate the comments made by Daungsupawong and Wiwanitkit (2025) and would like to clarify several key aspects of our study. Our primary objective was to evaluate reliable qPCR internal controls suitable for use in the reproductive tract, liver, and immune tissues and cells of adult male turkey breeders, including healthy individuals and those affected by Yellow Semen Syndrome (YSS). It is important to emphasize that our research focused solely on turkeys, not chickens, as was mentioned in comments written by Daungsupawong and Wiwanitkit (2025).

We are aware that the number of toms used in our experiment was rather limited, primarily due to restricted access to YSS birds within the specific age range of 32–36 weeks. It should be emphasized that all birds used in this experiment were kept under identical environmental conditions, and all sample collections were conducted using standardized protocols by the same qualified team to minimize variability. Due to the limited number of observations, this study was published as a Research Note.

It is worth mentioning that the issues of bird selection and sample size were thoroughly discussed during the peer-review process. We believe that the reference genes recommended by Drzewiecka et al. (2025) may be considered first-choice candidate reference genes for molecular biology experiments investigating reproductive, metabolic, and immune mechanisms in male breeder turkeys, especially in relation to YSS occurrence in commercial flocks.

## Declaration of competing interest

The authors declare that they have no known competing financial interests or personal relationships that could have appeared to influence the work reported in this paper.
